# Building a bonfire that remains stoked: sustainment of a contingency management intervention developed through collaborative design

**DOI:** 10.1186/s13011-015-0027-0

**Published:** 2015-08-06

**Authors:** Bryan Hartzler

**Affiliations:** Alcohol & Drug Abuse Institute, University of Washington, Box 354805, , 1107 NE 45th Street, Suite 120, Seattle, WA 98105-4631 USA

**Keywords:** Contingency management, Collaborative design, Behavior therapy dissemination

## Abstract

**Background:**

Community dissemination of empirically-supported behavior therapies is fostered by collaborative design, a joint process pooling expertise of purveyors and treatment personnel to contextualize a therapy for sustainable use. The adaptability of contingency management renders it an exemplary therapy to model this collaborative design process.

**Methods:**

At conclusion of an implementation/effectiveness hybrid trial conducted at an opiate treatment program, a group elicitation interview was conducted with the setting’s five managerial staff to cull qualitative impressions of a collaboratively-designed contingency management intervention after 90 days of provisional implementation in the setting. Two independent raters reviewed the audio-recording and conducted a phenomenological narrative analysis, extracting themes and selecting excerpts to correspond with innovation attributes (i.e., relative advantage, compatibility, complexity, trialability, observability) of a well-known implementation science framework.

**Results:**

This qualitative analysis suggested the intervention was regarded as: 1) cost-effective and clinically useful relative to prior practices, 2) a strong fit with existing service structure and staffing resources, 3) procedurally uncomplicated, with staff consistently implementing it as intended, 4) providing site-specific data to sufficiently inform decisions about its sustainment, and 5) offering palpable benefits to staff-patient interactions.

**Conclusions:**

The current work complements prior reports of positive implementation outcomes and intervention effectiveness for the parent trial, mapping qualitative managerial accounts of this contingency management intervention to a set of attributes thought to influence the speed and effectiveness with which an innovative practice is disseminated. Findings support the incorporation of collaborative design processes in future efforts to transport contingency management to the addiction treatment community.

## Background

Persistent calls to bridge science-to-practice gaps underscore a dilemma of dissemination for contingency management (CM), a behavior therapy principally utilizing operant conditioning principles to shape treatment-adherent behavior [1]. Strong empirical support exists for reliability of therapeutic benefits with substance users across a range of diverse CM methods [2–4]. Further, a widely-promoted prized-based CM method [5] demonstrated community effectiveness in multi-site studies conducted via NIDA’s Clinical Trials Network (CTN) [6, 7]. Nevertheless, issues of cost, logistical compatibility, and philosophical incongruence are common points of reticence toward CM by the addiction treatment community [8–10]. These concerns appear to weigh heavily on stakeholders in many settings, as evidenced by the 12 % rate of CM sustainment among CTN-affiliate community treatment programs in the years following these CTN trials [11]. Skepticism about the pragmatic utility of CM is likely perpetuated by subsequent published reports detailing procedural challenges with CM methods that led to their discontinuance in addiction settings in which implementation was attempted [12, 13]. This collective evidence suggests that CM holds great potential as a behavior therapy, but also that its effective dissemination to the addiction treatment community is likely to require a thoughtful, and perhaps more flexible approach.

Thoughtful approaches to CM dissemination may draw upon existing knowledge from transdisciplinary models of how innovative products are brought into routine use by a targeted community of consumers. A widely-known model, Rogers’ Diffusion of Innovations [14], offers a conceptual framework based on a half-century of research whereby a set of innovation attributes were identified that predict the speed and quality of adoption. With respect to disseminating CM to addiction settings, these attributes reflect: 1) relative advantage, or the extent to which CM is an improvement in cost- or clinical effectiveness over therapeutic practices previously in place in such settings, 2) compatibility, or the extent to which CM fits well with setting needs, interests, and values, 3) complexity, or the extent to which personnel in addiction settings (as agents of implementation) are able to both understand CM concepts and competently implement necessary procedures, 4) trialability, or the extent to which addiction settings can experiment with CM and evaluate its utility in their setting, and 5) observability, or the extent to which the consequent therapeutic impacts are palpable to addiction setting staff and their CM-exposed patients. Two decades ago, Rogers discussed issues of innovation adoption as relate to drug abuse prevention programs, citing the salience of several of the aforementioned innovation attributes for effective program dissemination [15]. Though the subsequent extant literature contains no application of these attributes from the *Diffusion of Innovations* framework specifically to CM dissemination, they nonetheless offer a useful conceptual background that may guide qualitative inquiry.

In specifying core tenets of CM, Petry [16] notes the identification of an observable target behavior, timely provision of tangible reinforcers upon its observance, and withholding of reinforcement in its absence. These tenets outline necessary features of any well-conceived CM intervention, yet also highlight a flexibility inherent in CM that makes it—unlike many behavior therapies—highly adaptive to context [17]. Specifically, these core tenets do not dictate need for standardization of other, malleable intervention features such as the targeted patient group or clinical behavior, tangible items to be made available as reinforcers, or specific schedule (i.e., frequency, duration) of reinforcement opportunities. Consequently, such malleable intervention features present opportunity for CM purveyors—or those who promote, design, and train others to use CM approaches—to tailor interventions to the specific needs, interests, and resources of individual addiction treatment settings. This inherent flexibility may foster more effective CM dissemination if purveyors engage their community partners in *collaborative intervention design*, a process that pools conceptual expertise of the purveyor and contextual insights from setting leadership. This approach to intervention design harkens back to early notions of technology transfer in the addiction field [18], in which the subjectivity of therapy concepts was highlighted, need for contextual adaptation was recognized, and creative synthesis of ideas from both the developers and consumers of a given behavior therapy was outlined as a collaborative process.

Collaborative intervention design is evident in an implementation/effectiveness hybrid trial, which evaluated the utility of a CM intervention at a community-based opiate treatment program (OTP) [19]. In preparation for the trial, a CM purveyor introduced the aforementioned core CM tenets to OTP leadership, after which the setting director was invited to define a set of malleable intervention features (full intervention design process detailed in *Methods*). The trial was consistent in scope with a Curran and colleagues’ [20] implementation/effectiveness hybrid ‘type 3’ design, with formal testing of implementation strategies for an empirically-validated behavior therapy and secondary evaluation of corresponding clinical effectiveness. A number of salient trial design features were included, in addition to the focal, collaboratively-designed CM intervention. Trial design features pertaining to OTP staff included recruitment for voluntary attendance of a CM training process, completion of serial training outcome assessments (prior to, just after, and three months following training), and post-training provisional implementation of the CM intervention with eligible patients on their caseload over a 90-day period. Additional trial design features included independent chart review of CM-exposed patients and comparison to those of a historical control patient group, and a group elicitation interview at trial conclusion wherein managerial staff offered qualitative opinions about setting implementation experiences. Previously-reported trial outcomes include: 1) recruitment of 80 + % of eligible OTP staff for trial participation, 2) robust, durable impacts of training on the CM fidelity, knowledge, and adoption readiness of OTP staff, 3) documented adoption among all trained staff who had opportunity to deliver the CM intervention during 90-day provisional implementation, 4) significant clinical effects (*d* = .45–.53) on targeted outcomes among CM-exposed patients, and 5) qualitative evidence of setting enthusiasm for post-trial sustainment of the CM intervention [19].

The current report expands upon this last, most intriguing trial outcome. In context of a group elicitation interview conducted with managerial OTP staff at trial conclusion, reactions to the collaboratively-designed CM intervention were elicited along with discussion of its prospects for post-trial sustainability in the setting. Reactions and discussion of this set of managerial OTP staff at that time now appear particularly salient, given subsequent setting decision to incorporate the CM intervention among its routine clinical service provisions. As of this writing (two years later), the CM intervention remains in routine use at the OTP. This example of successful CM dissemination amplifies the apparent utility of the collaborative design process, given reliable reports from setting leadership of two-year sustainment of the resulting CM intervention. The current report synthesizes managerial staff sentiments elicited at conclusion of the parent trial, characterizing their more immediate yet experience-based impressions of the CM intervention according to the aforementioned attributes in Rogers’ *Diffusion of Innovations* framework [14].

## Methods

The University of Washington Institutional Review Board approved all procedures of the parent trial, which are comprehensively described elsewhere [19]. The current report presents a qualitative analysis of remarks and discussion offered by managerial OTP staff during a group elicitation interview conducted as a 90-day period of provisional implementation drew to a close at trial conclusion. Prominent features of the parent trial design are summarized below.

### Parent trial design

The parent trial modeled an implementation/effectiveness hybrid ‘type 3’design [20], and evaluated a set of implementation strategies (i.e., collaborative intervention design, skills-based staff training, identification of on-site implementation leaders) at an OTP for a CM intervention delivered by its staff and supported by an existing operating budget [19]. Trial features included: 1) collaborative design of a CM intervention, 2) recruitment of OTP staff to therefore attend a 16-h training and complete serial outcome assessments prior to, just after, and three months following training, 3) opportunity for CM-trained staff to implement the intervention with their eligible patients for 90 days, and 4) independent chart review of CM-exposed vs. matched historical control patients. As pertains to this report, the parent trial included a group elicitation interview conducted at trial conclusion with the OTP’s five managerial staff to elicit experience-informed impressions of the CM intervention and discussion of its prospects for post-trial sustainment. Managerial sentiments offered in this interview were subject to narrative analysis, conceptually guided by five innovation attributes outlined in *Diffusion of Innovations* [14].

### Clinical setting

This private, non-profit OTP is located in an urban area of a large city in the northwestern United States, and has been providing medication-assisted treatment for opiate dependence for more than four decades. The OTP maintains a census of roughly 1000 patients, each receiving agonist medication, individual and group therapy, and case management. Documentation of this range of services is facilitated by the use of a comprehensive electronic medical records system. Regarding staff recruitment, 19 of the 22 direct-care staff consented to participate in the parent trial (the others were invited to voluntarily attend training sessions and implement CM with patients, but did not complete serial training outcome assessments). Age ranged from 27–88 years (M = 59.32, S.D. = 12.73), and most (89 %) were female. Racial composition was 79 % Caucasian, 16 % Multi-Racial, and 5 % Native American. As for educational attainment, 58 % had a master’s degree, 26 % had a bachelor’s degree, and 16 % had an associate’s degree. With respect to employment duration, many were long-tenured (M = 12.24 years, S.D. = 9.72).

With respect to organizational data on the OTP, participating staff completed the Survey of Organizational Functioning [SOF[21]] at trial outset. The SOF contains statements about one’s workplace, which individual staff members rated on five-point scales (1 = Strongly Disagree, 5 = Strongly Agree). The SOF includes four broad domains of organizational functioning, which encompass eighteen subscales. These are: organizational motivation for change (program needs, training needs, pressures for change subscales), resources (office, staffing, training, computer, e-cmu subscales), staff attributes (growth, efficacy, influence, adaptability subscales), and organizational climate (mission, cohesion, autonomy, communication, stress, change subscales). Means for the 18 SOF subscale scores were computed for the current sample, and compared to published norms based on 163 addiction treatment programs in the United States [22]. Applying a criterion of +/−.75 standard deviation from published SOF norms, the OTP was ‘representative’ on 15 of 18 subscales. Exceptions were in staff-report of computer resources (+1.2 S.D.), e-cmu resources (+.9 S.D.), and organizational communication (+.8 S.D.) subscales. Thus, OTP staff saw their workplace as particularly well-resourced in terms of computers and internet/email access, and strongly supportive of maintaining channels of inter-staff communication.

### Implementation strategies

A set of three implementation strategies were tested in the parent trial. These were the collaborative design of the CM intervention, skills-based training for staff, and identification of on-site implementation leaders to support provisional CM implementation (each outlined below).

#### Collaborative intervention design

After a purveyor-led orientation to core CM tenets, the setting director was invited to define the following malleable intervention features: 1) new enrollees as a target group, 2) attendance of weekly counseling visits as a target behavior, 3) low-cost gift cards to local vendors as reinforcers, and 4) a voucher-based ‘point-system.’ Notably, the setting director envisioned a staff-delivered intervention, and this (as well as the described features) was a good fit for the setting mission, service structure, and fiscal constraints. More specifically, a pre-existing mission of the OTP was to enhance its medication-assisted treatment via staff delivery of therapeutic services. Further, the service structure was idiographic, with newly-enrolled patients assigned to specific staff members with whom they were expected to attend weekly counseling visits. Poor attendance rates prompted the targeting of new enrollees and their counseling visits. Regarding its operating budget, monthly enrollment of 35–40 new patients at the OTP imposed some fiscal constraints as did the director’s stipulation that any CM implementation not adversely affect its capacity to provide other services-as-usual. Accordingly, the setting director advocated that staff monitor the target behavior, track points, and deliver reinforcers amidst usual care in counseling visits. With these intervention features in place, the purveyor devised a reinforcement schedule in which patients would earn points at attended visits to accumulate or be exchanged for reinforcers. To enrich likely clinical impacts, priming and escalation features were included such that bonus points were earned at initial and consecutively-attended visits. The purveyor and setting director conjointly reviewed the full intervention design, and the setting director formally approved it for provisional use at the OTP.

#### Skills-focused training

Two psychologists, each familiar with OTP settings, facilitated a 16-h CM training process for setting staff. The training structure and content were informed by community preferences for temporally-distributed sessions guided by experientially-based, skills-focused curricula [23]. Accordingly, the training was distributed as four weekly half-day group sessions, each occurring in a large on-site group room enabling trainer demonstrations and dyadic role-plays focused on behavioral rehearsal of specific intervention delivery skills. Focal intervention delivery skills corresponded with those outlined by a previously-validated fidelity instrument [24]. Training emphasized active learning strategies, such that for each individual CM delivery skill domain: 1) a brief conceptual rationale was outlined, 2) the trainers each provided a live demonstration of the skill in a contextualized role-play, and 3) staff members were paired to complete a similar dyadic role-play activity as behavioral rehearsal during which there were opportunities for timely provision of performance-based trainer feedback.

#### Identification of on-site implementation leaders

Setting preparation for implementation was augmented through a 30-min consultative planning meeting before each of the four staff training sessions. These meetings were attended by the CM purveyor, and the five managerial staff at the OTP: its executive director, deputy executive director, treatment director, assistant treatment director, and special projects officer. In the initial planning meeting, the OTP director identified two on-site implementation leaders who became responsible for preparatory activities (e.g., devising reinforcer purchasing/accounting systems, modifying electronic medical record system to enable CM-related staff notation) in advance of setting implementation. At conclusion of the staff training, these on-site implementation leaders provided local day-to-day oversight of staff during the provisional implementation period. Notably, local oversight was integrated into the setting’s *supervision-as-usual* practices, which included semi-weekly individual case review and weekly staff meetings. Consistent with phased therapy implementation models [25, 26], this occurred in conjunction with continued phone/email availability of consultative purveyor support on an as-needed basis for setting leadership and staff.

### Qualitative analytic evaluation

Upon conclusion of the trial implementation period, the purveyor led an audio-recorded 60-min group elicitation interview with the five managerial OTP staff. Prior to recording, each of these managerial staff provided informed consent. The interview structure was flexible, with a focus through open discussion on setting implementation experiences to date, impressions of the CM intervention, and interest in post-trial sustainment. Audio-recording of the interview was transcribed in its entirety, and conjointly reviewed by two raters in a phenomenological narrative analysis. Accordingly, managerial staff sentiments were treated as ‘windows into the lived experience’ [27] of provisional setting implementation of the collaboratively-designed CM intervention at this OTP. Raters compiled managerial staff sentiments corresponding with each of Rogers’ five innovation attributes [14], and then for each of these attributes selected one excerpt contributed by each of the five managerial staff members for inclusion in this report.

## Results

### Relative advantage

Managerial views of the collaboratively-designed CM intervention were generally quite positive, and this was reflected in sentiments regarding its advantages relative to prior practices in the OTP setting. In discussing these, prominent themes were managerial impressions of the enhanced therapeutic benefit and cost-effectiveness of the CM intervention. One sentiment cited initial reticence about this organizational undertaking, but also attenuation as those in the setting accumulated exposure via training and procedural experience during provisional implementation. Another sentiment noted the administrative burden of adding CM procedures to busy clinical interactions early in treatment, though framed this as worthwhile from a cost-benefit perspective.

“We’re going to keep doing this because it’s better for our patients. We weren’t going to invest

in something that didn’t give us some return. But [the CM intervention] gives us that return.

We’re going forward with this, it’s in the treatment manual and will continue to be part of the

services we provide here”.

“My hope was to better engage clients, like ‘we know this takes effort for you, and we recognize

it.’ There’s definitely therapeutic benefit—that’s what I’ve heard from our staff and the patients”.

“Though I don’t see the clients much, I touch base with the counselors when distributing the gift

cards. I’m looking forward to seeing the reported outcomes, to see if counseling attendance rate

improved like we hoped…….because people here seem like they’re enjoying this more”.

“Admittedly, I went into this with trepidation. Asking my already-stressed counselors to add

tasks to their early treatment interactions….for the organization to go in a new direction that may

or may not bear fruit, what would it do to morale? It was a leap of faith…..but one that improved

our services for these patients. So, I have confidence about [the CM intervention] going forward”.

“Well, as for challenges that [the CM intervention] presents…it is an extra component added to

an already loaded initial treatment burden that counselors have with folks coming into treatment.

So, that is an extra burden. But it seems to be worth it to staff, I hear good things from them about how rapport with new patients is better now”.

### Compatibility

In discussing the compatibility of the CM intervention for this OTP setting, managerial sentiments generally confirmed the intent of the collaboration intervention design process. The prominent theme was that the resulting intervention was a strong fit within the existing clinic infrastructure. Individual sentiments varied somewhat with respect to the emphasis placed on intervention compatibility with fiscal, logistical, or philosophical aspects of the setting. Each aspect of this existing setting infrastructure was reflected in multiple managerial sentiments.

“We had the right people in place, and [the CM intervention] seemed like the right thing to do for

our clinic. So all of my anxiety was eliminated, and I had confidence about how it would fit in here

and go forward”.

“The counselors, they seek these folks every week anyway, and deliver [the CM intervention] in

the context of a session we already pay staff time for. So….. there’s no added cost there, and the

administration time is fairly trivial”.

“The timing matches a point when patients’ treatment changes anyway….concluding as counseling frequency goes down and most patients are becoming stable. It’s really well-matched to the layout of our program”.

“The high need of people in this initial phase of treatment—it’s a real intense time for counselors

and patients, so to add something to that…it was important that it fit with what we’re trying to do,

which is to engage them. And it seems to do that”.

“It is a little more work…….it’s another piece of paper to fill out, another thing [for staff to check

off the list. My sense is that more people enjoy it rather than find it cumbersome”.

### Complexity

In interview discussion of the CM intervention’s complexity, a prominent theme was how simplicity in its design had promoted more consistent, clinically-useful implementation by staff. Further, this simplicity was suggested to have facilitated effective navigation of what challenges arose, and even enabled subsequent training of a newly-hired counselor who then contributed to setting implementation efforts. One sentiment noted the extra work involved in preparation for provisional implementation, and others noted desire or suggestions to further improve procedural efficiency. Notably, the magnitude of these voiced concerns typically paled relative to perceived benefits of clinic implementation of the CM intervention.

“In terms of the logistics involved, we’ve come up with solutions for just about everything that’s

come up. The implementation [of the CM intervention] does not need to be all that sophisticated to be done successfully”.

“Many other [CM approaches] would be too complicated to pull off in a consistent way. This

was do-able enough that we even later trained a new staff member who then used [the CM

intervention] with her whole caseload”.

“What made [the CM intervention] manageable was it was circumscribed in scope, and we had

two point-people that all questions could be directed to. That was critical”.

“The initial push to get ready for implementation took a bit of work, and mild concerns came up

in a client situation we didn’t anticipate. But, overall, it’s gone well”.

“I would advocate that, in looking ahead to using this in the future, we take some time to review

the point-system and consider if some small adaptations might be useful. The bonus points may

complicate this for some folks”.

### Trialability

With respect to intervention trialability, managerial sentiments seemed to suggest that the setting’s direct experiences during the 90-day period of provisional implementation had provided useful lessons. Some sentiments re-iterated how this experience had offered a chance to gather site-specific evidence of clinical effectiveness as well as to gauge staff motivation for continuing CM implementation thereafter. Other sentiments identified setting resources that had facilitated provisional implementation experiences. Still other sentiments looked ahead to the prospect of intervention sustainment. One sentiment focused on areas to target to improve future efficiency, whereas another identified additional staffing resources needed to support internal evaluation.

“Most of the counselors are interested in continuing with [implementing the CM intervention]. If people hated it, that would be different. But that’s not the case here. Assuming that the data show

positive effects, we’re all inclined to continue with this”.

“It’s one thing to say ‘the literature suggests this, that, or the other works,’ and it’s another thing

altogether for us to now have the experience of having it actually happen”.

“We’ve got an electronic record system where staff can grab patient information quickly, so that

made a big difference in terms of accessing what they needed, and for documentation”.

“To implement this long-term, we’ll need someone to track the data—to see how we’re doing. We

hired a person who can do that for us here, but it does reflect an additional [staffing] cost”.

“So…I would say that our biggest challenges have been tracking, and getting ample supply of the

gift cards out to the counselors in advance. Our system has been good, but I think it could still be

improved in terms of its efficiency”.

### Observability

As for observability, managerial sentiments suggested that beneficial impacts of the CM intervention for both OTP staff and patients had clearly been palpable. Many of these comments derived out of direct managerial discussions with staff in clinical supervision, staff meetings, or other common points of conversation in the milieu. One sentiment noted the added time required of CM procedures, but framed this as clinically worthwhile given the larger benefits of increased treatment adherence by patients. Other sentiments noted an enthusiasm for the CM intervention that had been observed among CM-exposed patients, as well as the utility of eliciting patient feedback about ways the setting might further enhance its appeal.

“I was really pleased to see so many of the counselors participate…in the training and then using

it with patients. They’ve done a good job of implementing it and are pretty positive about it.”

“The staff ….they really like acknowledging and positively reinforcing patients in this way.”

“The majority of [OTP staff] enjoy it…..and the clients really like it. Counselors actually call me

in when their clients are there who say ‘I really like this program.’ I hear that kind of thing a lot.”

“[CM intervention procedures] may take away five minutes of a session….but if you have people

coming in more regularly you get to focus on things other than noncompliance”.

“Another good thing we got was [patient] feedback to include other incentives, like lock-boxes for

take-home medication doses. That was a great patient suggestion, and we can offer things like that

as additional incentives”.

## Discussion

By way of a conceptually-driven qualitative analysis of innovation attributes, this report presents experience-informed managerial impressions of a contextualized CM intervention after its 90-day provisional implementation in a community-based addiction setting. The intervention, developed through a collaborative design process that pooled the conceptual expertise of a CM purveyor and contextual insights of the setting director, was subsequently sustained among the setting’s routine clinical service provisions over a multi-year period. Narrative analysis of a group elicitation interview with managerial setting staff suggests the collaboratively-designed intervention was regarded as: 1) cost-effective and clinically-useful relative to prior practices, 2) a fit with the setting’s operating budget, staffing and service structure, and philosophical mission, 3) procedurally uncomplicated for setting staff to implement, 4) providing site-specific data to sufficiently inform setting decisions about its sustainment, and 5) offering palpable benefits to staff-patient interactions. This qualitative data supplements prior report of a host of encouraging outcomes of this parent implementation/effectiveness hybrid trial [19]. This successful example of CM dissemination may help to inform broader translational efforts for this behavior therapy.

A salient consideration in translating findings of implementation trials to broad therapy dissemination efforts is the representativeness of the involved settings [28]. This takes on added significance for the current trial, given its single-site design. A breadth of organizational data for the involved setting was available, and the bulk of this data—encompassing the OTP’s readiness for change, resources, staff attributes, and organizational climate—suggested it to be reasonably representative of the domestic addiction treatment community [22]. Specific areas of exception were its greater staff-perceived computer/e-communication resources and support of inter-staff channels for communication. It is not difficult to imagine these inter-related characteristics may have aided setting implementation of this CM intervention, as can be inferred from included managerial sentiments. Further, it is consistent with prior research where similar characteristics domains predict conductive attitudes toward CM [29] and other empirically-supported practices [30]. While a potential caveat to the generalizability of current findings, this information is useful in terms of specifying resources likely to facilitate systemic therapy implementation. An additional setting characteristic with limited representativeness was the tenure of staff employment, which was lengthier at this OTP than in many community addiction settings [31, 32]. The relative absence of staff turnover at this OTP, and consequent challenges that would otherwise have been posed in perpetual training of new staff [33], certainly may have facilitated multi-year sustainment of the focal CM intervention. Of course, any such possible beneficial influences of employment tenure in addiction settings should be considered alongside extant literature where the evidence of its influence on learning and adoption of new therapeutic practices is equivocal [34, 35].

These findings add to a burgeoning literature on attempts to implement CM approaches in addiction treatment settings. Notably, the collaborative intervention design process outlined herein overlaps in both philosophy and procedure with that of a multisite dissemination effort successfully undertaken in the New York-based Health and Hospitals Corporation by Kellogg and colleagues [36]. Similarities between these two successful dissemination efforts include that: 1) initial purveyor-led orientation to core CM principles was provided, 2) substantial input from setting stakeholders was solicited in the design of setting-specific CM interventions, 3) dominion over the end-product and onset of implementation was left to the treatment settings, and 4) data on clinical outcomes was evaluated, with timely purveyor feedback offered to setting leaders to inform decisions about sustainment. Thus, these may be a set of core conditions that facilitate effective dissemination of CM to community-based addiction settings, particularly given their absence in prescriptive technology transfer processes the eventuated in setting discontinuance of CM approaches [12, 13]. Still, challenges remain to broader understanding of how behavior therapy implementation is best sustained. As reviewed by Stirman and colleagues [37], these include lack of consensus in terminology, limited domains of measurement, poor appreciation for interactive influences, and an over-reliance on retrospective and/or naturalistic study designs.

This work carries several caveats. Prominent among these is the single-site design of the parent trial, for which generalizability of findings is difficult to know. In most organizational functioning domains, the OTP was reasonably representative of settings in the U.S. addiction treatment community. Exceptions were its considerable computer and e-cmu resources, strong support of inter-staff communication, and lengthy tenure of employment among participating staff. While posing threats to the generalizability of study findings, these setting attributes merit further attention as facilitating influences for CM implementation. Other caveats of the parent trial were self-selection bias inherent in voluntary participation of OTP staff, a somewhat brief period of provisional implementation, and absence of formal data collection to document post-trial sustainment. Regarding the latter, the author posed consistent inquiries to setting leadership at biannual intervals, and his continued local working relationship with this OTP offers assurance of the veracity of those informal setting reports. Nevertheless, such reports do not specifically address issues of continuing staff fidelity or clinical effectiveness in the setting. Other caveats for current findings concern sampling and data collection procedures for the elicitation interview. Recruitment of all five managerial staff effectively saturated this organizational stratum of the OTP, but precluded input from its direct-care staff and patients about provisional implementation experiences. Relatedly, the use of a group elicitation interview left open possible influences of demand characteristics, as managerial sentiments were offered in the presence of peers. Further, collection of this data at a single point in time negated opportunity to track possible changes in opinions over time as implementation experience further accumulated. Finally, and as with any qualitative research, the interpretive nature of data collection and analysis is a potential concern.

## Conclusions

Caveats notwithstanding, the principal contribution of this work is its qualitative mapping of managerial impressions—gathered amidst successful adoption of a collaboratively-designed CM intervention—to concepts of a well-known implementation framework. What suggestions may be drawn from these findings to aid therapy purveyors in prospective dissemination of CM? One is that purveyors strongly consider inviting community partners to help sculpt interventions to be delivered at their facilities. Collaborative intervention design offers a tangible vehicle for doing so. Its pooling of conceptual expertise and contextual insights may uncover new edges of therapy innovation, a notion broadly consistent with other successful efforts to disseminate CM to addiction settings [36, 38]. A 2^nd^ suggestion for purveyors is that they move past presumptive notions of standardized therapy implementation in clinical practice as an eventual goal for all behavior therapies [39, 40]. A growing scientific consensus posits flexible setting adaptation as necessary to guide behavior therapy dissemination [41, 42]. As this work evidences, circumstances exist where contextualization of CM is precisely what is needed for successful implementation. A 3^rd^ suggestion is that purveyors utilize implementation science models to inform their work. Many applicable models exist [25, 43–45] beyond the Diffusion of Innovations framework [14] that guided this qualitative analysis. As for current findings, these collective managerial impressions suggest a bonfire of CM implementation may remain stoked if community treatment partners are invited to tailor identified intervention features to fit contextual interests, needs and resources.

## Abbreviations

CM: Contingency management
OTP: Opiate treatment program
